# Sedentary lifestyle of university students is detrimental to the thoracic spine in men and to the lumbar spine in women

**DOI:** 10.1371/journal.pone.0288553

**Published:** 2023-12-05

**Authors:** Alena Cepková, Erika Zemková, Ľubomír Šooš, Marián Uvaček, José M. Muyor

**Affiliations:** 1 Faculty of Mechanical Engineering, Slovak University of Technology, Bratislava, Slovakia; 2 Faculty of Physical Education and Sport, Comenius University in Bratislava, Bratislava, Slovakia; 3 Faculty of Health Sciences, University of Ss. Cyril and Methodius in Trnava, Trnava, Slovakia; 4 Health Research Centre, University of Almería, Almería, Spain; Opole University of Technology: Politechnika Opolska, POLAND

## Abstract

**Background:**

Sitting for long periods of time and lack of physical activity in young adults can cause postural deterioration leading to rapid onset of fatigue and increase the risk of back pain. We were interested in whether there are gender differences in spinal curvature among university students with a predominantly sedentary lifestyle.

**Methods:**

20 sedentary female (age 20 ± 0.73 years) and 39 sedentary male university students (age 20 ± 1.08 years) participated in this study. Their thoracic and lumbar curvatures were assessed while standing and sitting using a Spinal Mouse.

**Results:**

In standing, 80.0% of the females and 69.2% of the males had a neutral position of the thoracic spine (33.25° and 35.33°, respectively). However, more males, 30.8%, than females, 10.0%, had hyperkyphosis (54.27° and 47.0°, respectively). Hypokyphosis was found in 10.0% of the females (18.50°) and none in the males. Similarly, 90.0% of the females and 97.4% of the males had neutral position of the lumbar spine (-33.11° and -29.76°, respectively). Increased hyperlordosis was found in 10.0% of the females and 2.6% of the males (-41.0° and -50.0°, respectively). Hypolordosis was not detected in either females or males. In sitting, on the other hand, 70.0% of the females and only 33.3% of the males had a neutral position of the thoracic spine (30.20° and 30.62°, respectively). Increased hyperkyphosis was found in 46.2% of the males (59.76°) and none of the females. 30.0% of the females and 23.1% of the males had light hypokyphosis (47.50° and 46.67°, respectively). Similarly, 70.0% of the females and only 38.5% of the males had a neutral position of the lumbar spine (7.0° and 6.6°, respectively). 35.9% of the males and only 5.0% of the females had a light hypokyphosis (16.14° and 16.0°, respectively). Slightly increased hyperkyphosis was identified in 25.6% of the males and 25.0% of the females (23.9° and 22.5°, respectively).

**Conclusion:**

There are significant gender differences in spinal curvature. While in the thoracic spine it was to the detriment of the males when both standing and sitting, in the lumbar spine it is related to the females only when standing. It is therefore necessary to eliminate these spinal deviations in young adults induced by prolonged sitting during university courses by appropriate recovery modalities.

## Introduction

After starting their university studies, students acquire sedentary habits with a very low contribution of leisure activities [1, 2]. This causes reduced physical performance in young adults [3]. One consequence is lower back pain, which is associated with leisure-time physical inactivity [4]. This trend has been confirmed by a number of authors following the monitoring of university students`physical fitness o [5, 6]. Adults sit for eight hours daily and are on average active for 4 hours. This is a great disparity, which is reflected in their worsening physical fitness. The lack of physical activities and the predominance of a seated posture among students leads to an overloading of the same joint structures and the same muscle groups [7]. Motion passivity causes an insufficiency of the information coming into the central nervous system, which shares in the emergence of faulty motion stereotypes and muscle imbalance [8]. The main causes of muscle imbalance include hypokynesis, chronic loading above the limit set by muscle quality, asymmetric loading without sufficient compensation, and changes to the mobility stereotype, for example as a result of an injury or an illness [9].

Lack of movement and inappropriate physical activities, static overburdening, and unilateral loading affects posture [10]. Abnormal posture places strain on the ligaments and muscles, which may indirectly influence spinal curvature. Sitting for just 1 hour leads to increased spinal stiffness [11]. Thoracic mobility is reduced in individuals who spend >7 hours/day sitting and <150 min/week of physical activity [12]. There is a relationship between prolonged sitting and thoracic mobility, with >10° less mobility for sedentary than for physically active individuals [12]. Sitting also causes a reduction in lumbar lordosis and pelvic region, which could lead to a spinopelvic imbalance [13]. When sitting, the knees and hips are flexed, the pelvis rotates backward, and lumbar lordosis flattens [14]. A decrease of the trunk-thigh angle while sittting leads to flattening of the lumbar curve [15, 16]. Along with this, the lumbar intradiscal pressure increases [17, 18], which can contribute to the risk of developing back pain.

It is known that women are less active than men, thus they have significantly higher levels of sedentary behaviour [18]. However, the question remains as to whether there are gender differences in thoracic kyphosis and lumbar lordosis among young adults with a predominantly sedentary lifestyle.

## Materials and methods

### Participants

Two heterogeneous groups of randomly selected students of the mechanical engineering study program participated in the study: 39 males (age 20.0 ± 1.1 years, height 181.2 ± 6.9 cm, body mass 77.7 ± 12.3 kg, BMI 23.8 ± 1.6 kg/m^2^) and 20 females (age 20.0 ± 0.7 years, height 169.1 ± 4.2 cm, body mass 61.1 ± 5.8 kg, BMI 21.4 ± 1.4 kg/m^2^). The BMI of both groups is within the norm according to the WHO classification and does not affect their posture. A disparity between groups was due to a higher proportion of males (80%) than females (20%) of all university students.

Participants filled out the questionnaire that was related to basic demographic information, such as age, height, body mass, BMI, as well as to the inclusion criteria, including absence of pregnancy, absence of any regular sport, sedentary work for 8–10 hours a day, lack of history of spine surgery, absence of history of orthopedic disease in the past 5 years, no specific drug use for musculoskeletal or neurovascular disorders, no history of irreversible kyphosis or lordosis, and no history of scoliosis. They took part in compulsory optional physical education courses, once a week. While females participated in various types of aerobics, males played indoor soccer and floorball.

All participants were informed in advance about the course of the testing and verbally agreed to its conditions, with the participation of two witnesses, the investigators. The procedures followed were in accordance with the ethical standards for human experimentation outlined in the 1964 Declaration of Helsinki and its later amendments. The project was approved by the Ethics Committee of the Faculty of Physical Education and Sport of Comenius University in Bratislava (No. 4/2017).

### Assessment of spinal curvature

Testing was performed by a member of our research team with long-term experience in spinal mouse measurement together with an assistant. One measurement took an average of 30 minutes. The measurements were carried out according to standard practices, ensuring compliance with hygienic, safe, and discreet conditions. The men were unclothed on the upper part of their bodies, while the women wore a swimsuit top and sports pants. Participants were assessed using the same methodology in two different positions, utilizing a spinal mouse. The first set of measurements aimed to evaluate the spine in an upright standing position, while the second set focused on an upright sitting position.

A computer based electro-mechanical Spinal Mouse device was used to assess posture (Idiag, Fehraltdorf, Switzerland). The Spinal Mouse is a valid and reliable device for measuring global thoracic and lumbar curvature compared to radiographic techniques [19–21], documented by an intraclass correlation coefficients (ICC) greater than 0.8 and a standard error of measurement (SEM) of less than 4º for all spinal parameters evaluated [20].

Measurements were performed at the sagittal level in a relaxed standing and sitting position, in a random order. All measurements were taken on the same day, in the same environment and under similar conditions. There was a 5-minute rest between each measurement. The examiner palpated the starting point C7 and the upper part of the anal fold (end point) and marked them on the skin. The examiner passed the Spinal Mouse along the central axis of the spine (or slightly paravertebral in particularly thin individuals) from C7 to the upper part of the anal fold (about S3). In each position, the values of the thoracic (T1-2 to T11-12) and lumbar vertebrae (T12-L1 to the sacrum) were recorded. In the lumbar curve, negative values corresponded to lumbar lordosis (concavity of the back).

Rating positions included standing, where the participant stood in a relaxed position with the head erect, hands next to the body, knees extended, feet shoulder width apart; and sitting, where the participant sat on the edge of the chair, knees bent to a 90° angle with legs apart, not touching the ground, head slightly bowed, hands on knees.

**Fig 1:**
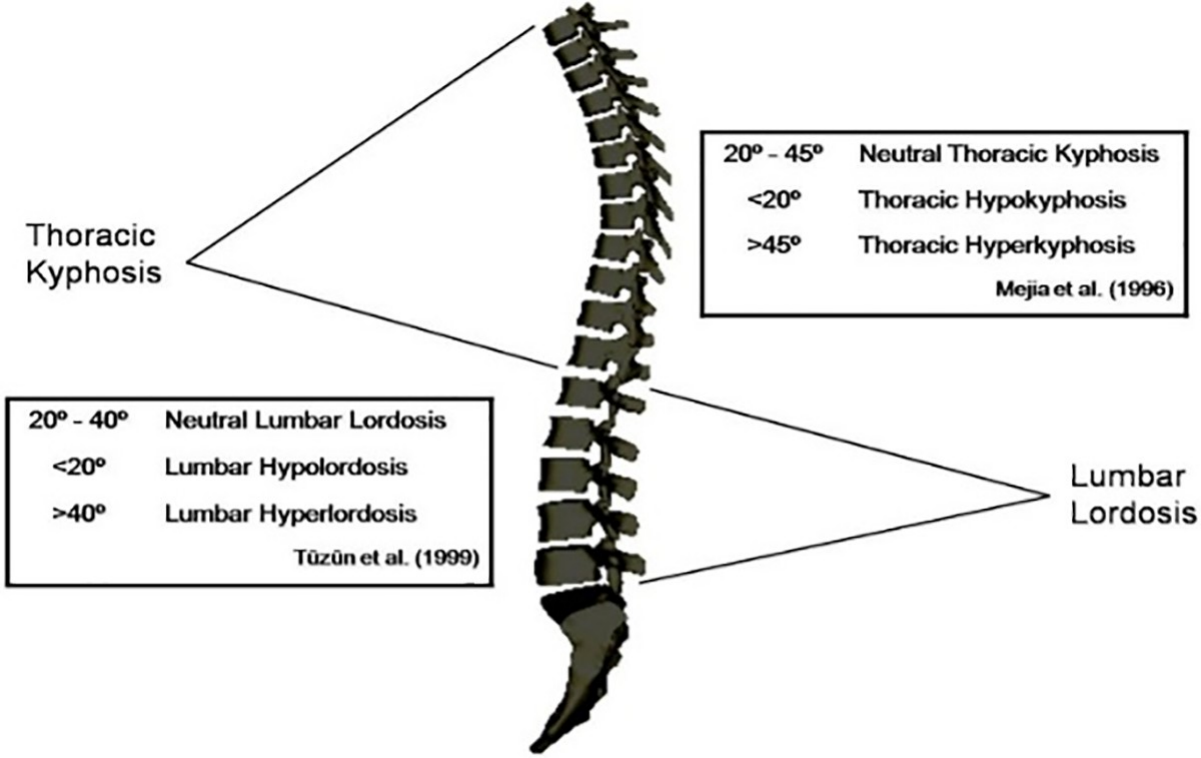
Classification of the spine when standing according to Mejia et al. [22] and Tüzün et al. [23].

When evaluating a standing posture using the Spinal Mouse, the classification of the thoracic spine according to Mejia et al. [22] was used (Fig 1). Values between 20° and 45° were accepted as neutral thoracic kyphosis, values less than 20° were considered thoracic hypokyphosis, and values greater than 45° were considered thoracic hyperkyphosis. For the evaluation of the lumbar spine, a classification according to Tüzün et al. [23] was used (Fig 1). In a standing position, the lumbar curve values between 20° and 40° were taken as neutral, values less than 20° were considered as hypolordotic, and values greater than 40° were considered as hyperlordotic.

**Fig 2:**
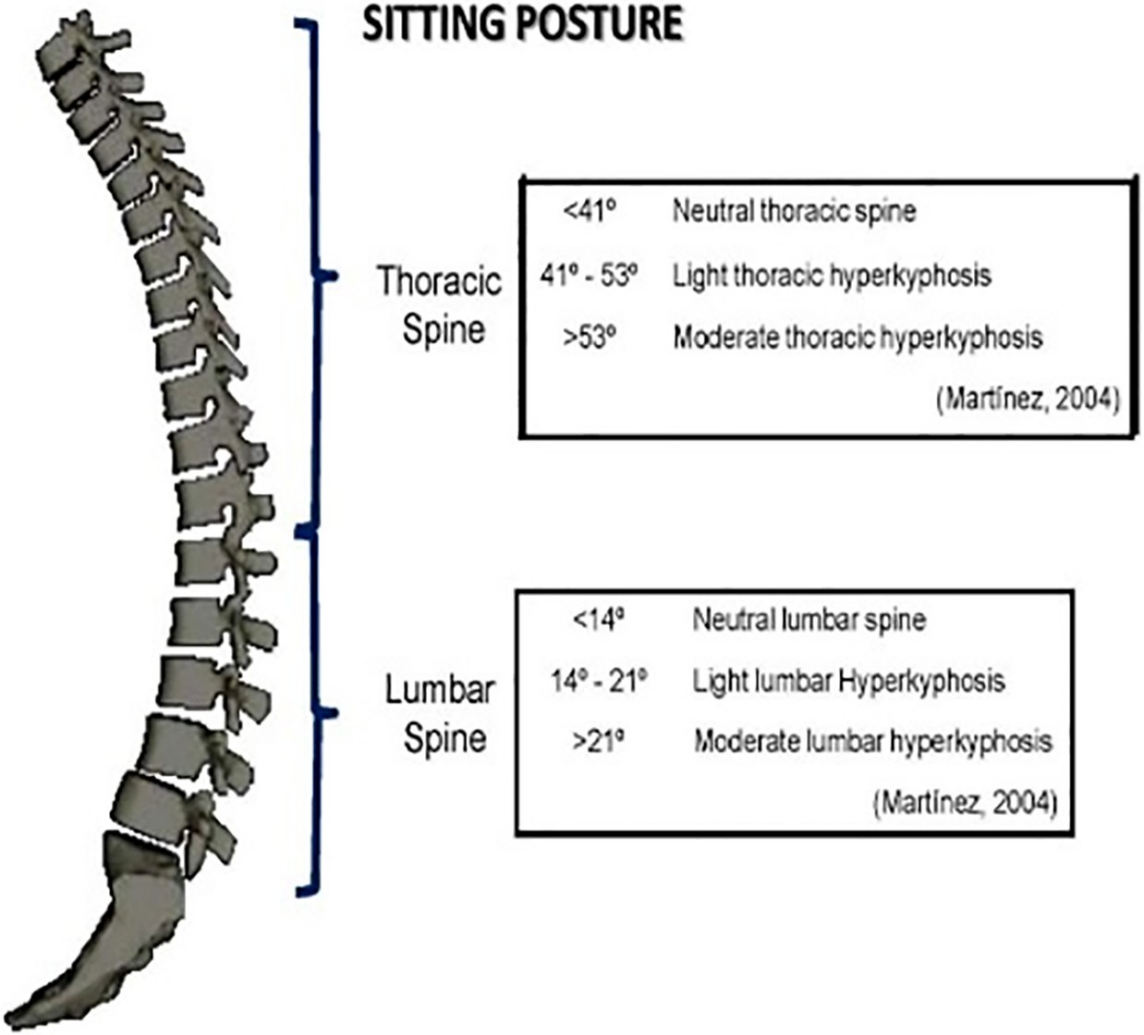
Classification of the spine when sitting according to Martinez [24].

When evaluating a sitting posture with the Spinal Mouse, the classification of the thoracic part of the spine according to Martinez [24] was used (Fig 2). Values less than 41° were accepted as a neutral thoracic spine, values from 41° to 53° were considered as light thoracic hyperkyphosis, and values over 53° were considered as increased light hyperkyphosis. A classification according to Martinez [24] was also used for evaluation of the lumbar part of the spine (Fig 2). In a sitting position, lumbar curve values of less than 14° were considered neutral, values from 14° to 21° were considered as light lumbar hyperkyphosis, and values over 21° were considered as increased light lumbar hyperkyphosis.

In the sitting position, the spine has different curvature values than in the standing position, which can furthermore indicate the possible risks of LBP.

### Statistical analysis

Data analyses were performed using the SPSS statistical program for Windows, version 18.0 (SPSS, Inc., Chicago, IL, USA). Descriptive statistics, including mean and standard deviations, were calculated for all variables. The hypotheses of normality and homogeneity of variance were analyzed using the Shapiro-Wilk test. An unpaired Student *t*-test was conducted to examine differences between females and males for all variables. The significance level was set at *p* < 0.05.

## Results

In a standing position, 80.0% of the female students had a neutral position of the thoracic spine, with a mean value of 33.25°. 69.2% of the male students had a neutral position of the thoracic spine, with a mean value of 35.33°. Hypokyphosis, with a mean value of 18.50°, was found in 10.0% of the female students, and none in the male students. Hyperkyphosis, with a mean value of 54.27°, was found in 30.8% of the male students, compared to only 10.0% of the female students, with a mean value of 47.0° (Table 1).

**Table 1:** Values of thoracic kyphosis and lumbar lordosis in a standing position for male and female students.

	Thoracic spine values while standing			Lumbar spine values while standing		
	20°–45°	< 20°	> 45°	20°–40°	< 20°	> 40°
	Neutral thoracic kyphosis	Thoracic hypokyphosis	Thoracic hyperkyphosis	Neutral lumbar lordosis	Lumbar hypolordosis	Lumbar hyperlordosis
**Females**	80.00%	10.00%	10.00%	90.00%	0.00%	10.00%
Mean	33.25°	18.50°	47.00°	-33.11°	0.00°	-41.00°
Min	25.00°	18.00°	46.00°	-25.00°	0.00°	-41.00°
Max	45.00°	19.00°	48.00°	-40.00°	0.00°	-41.00
**Males**	69.23%	0%	30.77%	97.44%	0%	2.56%
Mean	35.33°	0.00°	54.27°	-29.76°	0.00	-50.00°
Min	21.00°	0.00°	46.00°	-39.00°	0.00	-50.00°
Max	45.00°	0.00°	65.00°	-16.00°	0.00	-50.00°

Furthermore, 90.0% of the female students had a neutral position of the lumbar spine, with a mean value of -33.11°. Similarly, 97.4% of the male students had a neutral position of the lumbar spine, with a mean value of -29.76°. Hypolordosis was not detected in either the female or male students. Increased hyperlordosis, with a mean value of -41.0°, was found in 10.0% of the female students, and only in 2.6% of the male students, with a mean value of -50.0° (Table 1).

Between genders, significant differences in the thoracic part of the spine when standing were to the detriment of the male students, whilst in the lumbar part of the spine they were related to the detriment of the female students (Tables 2 and 3).

**Table 2:** Statistical analysis of the thoracic and lumbar spine values while standing in male and female students.

Thoracic spine values while standing			Lumbar spine values while standing	
	Females	Males	Females	Males
	(Col_1)	(Col_5)	(Col_2)	(Col_6)
Median	32	45	-35	-28.5
Minimum	19	25	-41	-37
Maximum	48	60	-25	-22
Range	29	35	16	15
Lower quartile	28	36.5	-37.5	-34.5
Upper quartile	37.5	50	-32	-23.5
Interquartile range	9.5	13.5	5.5	11

Col_1 shows thoracic spine values in the females while standing, Col_2 shows thoracic spine values in the males while standing, Col_5 shows lumbar spine values in the females while standing, Col_6 shows lumbar spine values in the males while standing

**Table 3:** Differences between genders in the thoracic and lumbar spine data measured in a standing position.

Contrast		Sig.	Difference	+/- Limits
Col_1	Col_5	*	-9.46538	5.49245
Col_2	Col_6	*	-3.61795	3.31012

* denotes a statistically significant difference (α = 0.05) between Col_1 (thoracic spine values in the females while standing) and Col_5 (lumbar spine values in the females while standing), Col_2 (thoracic spine values in the males while standing) and Col_6 (lumbar spine values in the males while standing)

In sitting, 70.0% of the female students had the thoracic part of the spine in a neutral position, with a mean value of 30.20°. However, only 33.3% of the male students had the thoracic part of the spine in a neutral position, with a mean value of 30.62°. Here we found a significant difference in comparison with standing, where 69.23% of the students had an approximately equal value of 35.33°. 30.0% of the female students had light hypokyphosis, with a mean value of 47.50°. About equal values of 46.67° were found in 23.1% of the male students. 46.2% of the male students had indications of mild hyperkyphosis, with a mean value of 59.76°, whereas not a single case of increased hyperkyphosis was indentified among the female students (Table 4).

**Table 4:** Values of thoracic kyphosis and lumbar lordosis in a seated position for male and female students.

	Thoracic spine values while sitting			Lumbar spine values while sitting		
	< 41°	41°–51°	> 53°	< 14°	14°–21°	> 21°
	Neutral thoracic spine	Light thoracic hypokyphosis	Mild thoracic hyperkyphosis	Neutral lumbar spine	Light lumbar hypokyphosis	Mild lumbar hyperkyphosis
**Females**	70.00%	30.00%	0%	70.00%	5.00%	25.00%
Mean	30.2°	47.5°	0	7.0°	16.0°	22.5°
Min	8.0°	42.0°	0	22.0°	16.0°	22.0°
Max	40.0°	51.0°	0	12.0°	16.0°	24.0°
**Males**	33.33%	23.08%	46.15%	38.46%	35.90%	25.64%
Mean	30.62°	46.67°	59.76°	6.6°	16.14°	23.9°
Min	22.0°	42.0°	50.0°	1.0°	14.0°	22.0°
Max	40.0°	51.0°	79.0°	12.0°	21.0°	27.0°

Furthermore, 70.0% of the female students and only 38.5% of the male students had a neutral position of the lumbar part of the spine, with a mean value of 7.0° and 6.6°, respectively. A light hypokyphosis, with a mean value of 16.14°, was found in 35.9% of the male students, and only in 5.0% of the female students, with a mean value of 16.0°. Slightly increased hyperkyphosis, with a mean value of 22.5°, was identified in 25.0% of the female students. Also 25.6% of the male students had slightly increased hyperkyphosis, with a mean value of 23.9° (Table 4).

Between the genders, there were significant differences in the thoracic part of the spine when sitting, to the detriment of the male students. However, no significant differences between genders were detected in the lumbar part of the spine while sitting (Tables 5 and 6).

**Table 5:** Statistical analysis of the thoracic and lumbar spine values while sitting in male and female students.

	Thoracic spine values while sitting		Lumbar spine values while sitting	
	Females	Males	Females	Males
	(Col_1)	(Col_5)	(Col_2)	(Col_6)
Median	38.5	55	8	13
Minimum	18	38	-22	-27
Maximum	49	68	24	23
Range	31	30	46	50
Lower quartile	27	44.5	-1.5	3
Upper quartile	44	60	17	19.5

Col_1 shows thoracic spine values in the females while sitting, Col_2 shows thoracic spine values in the males while sitting, Col_5 shows lumbar spine values in the females while sitting, Col_6 shows lumbar spine values in the males while sitting.

**Table 6:** Differences between genders in the thoracic and lumbar spine data measured in a seated position.

Contrast		Sig.	Difference	+/- Limits
Col_1	Col_5	*	-11.5256	7.12553
Col_2	Col_6		-4.11538	6.21936

* denotes a statistically significant difference (α = 0.05) between Col_1 (thoracic spine values in the females while sitting) and Col_5 (lumbar spine values in the females while sitting), Col_2 (thoracic spine values in the males while sitting) and Col_6 (lumbar spine values in the males while sitting).

## Discussion

This study revealed gender-specific differences in spinal curvature. In the thoracic spine they are to the detriment of men when standing and sitting, whereas in the lumbar part to the detriment of women when standing. Young university students, just like office workers, spend most of their time in a sedentary manner at schools and during their leisure time [25–27]. This sedentary lifestyle puts them at a permanent risk of developing future musculoskeletal problems. Sedentary individuals experience higher fatigue compared to physically active individuals, primarily due to prolonged sitting [28], which can be a major cause of back pain. Based on these facts, it is most likely that regular engagement in sports among university students would lead to improved posture, i.e. a lower percentage of men would have chest hyperkyphosis, and fewer women would have lumbar hyperlordosis.

Lack of exercise leads to joint contracture, joint narrowing, and muscle stiffness [29, 30]. Additionally, it increases antagonistic co-contraction for stability, resulting in increased spinal compression [31]. Fatigue of back muscles, loss of muscle mass in young healthy individuals aggravates their ability to modify their posture strategy and may lead to a similar posture strategy to that seen in patients with recurrent low back pain as postural demands increase [32–36]. At present, a trend of increasing functional breakdowns of the locomotor system is apparent. Among students, this arises from adaptation to a daily mobility regime in which the same muscle groups are overused, leading to faulty mobility stereotypes [37, 38]. Research confirms the occurrence of sexual dimorphism of shortened and weakened muscles with a trend towards a greater occurrence of shortened muscles among the male population and weakened ones among the females [39–41]. Differences in posture between men and women have been confirmed by several researchers [42–44]. It is clear that it is very important to perform compensatory exercises, stretching, targeted strengthening exercises and exercises aimed at training the correct movement stereotypes to reduce the onset of neck and low back pain [45–48]. As a result, muscular activation is more effective and also muscular strength is increased. This is because thoracic joint mobilization or self-stretching exercises for the spine improve limited movements of the spine, recover facet joint sliding, and normalize the articular capsule, thereby decreasing kyphosis and enhancing the flexibility of thoracic extension [49–51]. The results of the proposed exercise are reflected in the overall posture.

In our research, a higher incidence of hyperlordosis in female than male students was found. A study by Jankowicz-Szymańska et al. [52] demonstrated that hyperlordosis is characteristic for young female students, while hypolordosis is characteristic for older women. In general, an increase in thoracic kyphosis causes lumbar hyperlordosis to maintain sagittal balance [53]. It can be said that the higher incidence of hyperkyphosis in men may be a clinical sign of the presence of osteoporosis and a potentially modifiable risk factor for adverse health consequences [54].

Furthermore, a higher incidence of hyperkyphosis in male than in female students was identified in our study. According to Almujel et al. [55], the correct curvature of the spine correlates significantly with the strength of the back muscles. The relationship between lordosis and kyphosis is more recognized in achieving sagittal alignment [56–60]. The emphasis on spinopelvic harmony was first outlined by Dubousset [61], whose idea of a "cone of balance" described a specific position of the spine in standing that allowed the body to remain in balance with minimal muscle mass. Changes in one part of the spine can lead to unintentional changes in another area [57]. The observed negative relationship suggests that when either thoracic or lumbar curvature increases due to less muscle load, regional bone mineral density decreases [62]. Factors such as age and gender could, therefore, affect loading through changes in lordosis. At the same age, body height (BH) and body weight (BW) female spines are subjected to greater loads due to the associated smaller arm muscules and passive joint contributions [63]. Increased kyphosis and thus lordosis cause many problems, including back pain [64] and standing imbalance [65–67]. Prolonged sitting increases muscle fatigue and promotes an increase in existing spinal curvature, which worsens posture and can be a possible cause of back pain for both men and women. As part of low back pain prevention, we recommend taking short regular breaks and performing compensatory exercises while sitting and standing. In addition to exercises for the spine as a whole, attention should be paid to those able to eliminate thoracic kyphosis in male students and lumbar lordosis in female students.

The main limitation of this study is the small sample of female students, which is due to the overall low number of female students at the university of technology and the subjectivity of the assessment procedure. For this reason, these findings cannot be generalized to the broader community based on this study alone.

## Conclusions

There are significant gender differences in spinal curvature. While in the thoracic spine it was to the detriment of the males when both standing and sitting, in the lumbar spine it is related to the females only when standing. More specifically, the majority of the females and males had a neutral position of the thoracic spine (80.0% vs 69.2%) and the lumbar spine (90.0% vs 97.4%) while standing. However, more males than females had hyperkyphosis (30.8% vs 10.0%) and hypokyphosis (10.0% vs 0%), whilst hyperlordosis was found more in females than males (10.0% and 2.6%). On the other hand, more females than males had a neutral position of the thoracic spine (70.0% vs 33.3%) and the lumbar spine (70.0% vs 38.5%) while sitting. Thoracic hyperkyphosis was found only in males (46.2%), and hypokyphosis slightly more in females than males (30.0% vs 23.1%). More males than females showed lumbar hypokyphosis (35.9% vs 5.0%), whereas the occurrence of hyperkyphosis was comparable (25.6% vs 25.0%). These findings indicate that the sedentary lifestyle of university students is detrimental to the thoracic spine in men and to the lumbar spine in women. Therefore, it is necessary to eliminate these spinal deviations in young adults (thoracic kyphosis in males and lumbar lordosis in females) induced by prolonged sitting during university courses by appropriate recovery modalities.
